# The characteristics of the gut microbiota in patients with Kawasaki disease: a systematic review

**DOI:** 10.3389/fmicb.2025.1715478

**Published:** 2025-12-18

**Authors:** Hongbo Chen, Hanmin Liu, Lina Qiao, Yang Liu, Dan Yu, Zhiling Wang, Tao Wang, Weiran Li

**Affiliations:** 1 Department of Medical Genetics/Prenatal Diagnostic Center, West China Second University Hospital, Sichuan University, Chengdu, China; 2 Key Laboratory of Birth Defects and Related Diseases of Women and Children (Sichuan University), Ministry of Education, Chengdu, China; 3 Department of Pediatrics, West China Second University Hospital, Sichuan University, Chengdu, China; 4 Department of Pediatric Pulmonology and Immunology, West China Second University Hospital, Sichuan University, Chengdu, China; 5 NHC Key Laboratory of Chronobiology (Sichuan University), Chengdu, China; 6 The Joint Laboratory for Lung Development and Related Diseases of West China Second University Hospital, Sichuan University and School of Life Sciences of Fudan University, West China Institute of Women and Children’s Health, West China Second University Hospital, Sichuan University, Chengdu, China; 7 Sichuan Birth Defects Clinical Research Center, West China Second University Hospital, Sichuan University, Chengdu, China; 8 Department of Pediatric Pulmonology and Immunology, WCSUH-Tianfu, Sichuan Provincial Children’s Hospital, Sichuan University, Meishan, China

**Keywords:** gut microbiota, Kawasaki disease, Illumina sequencing, gut-vascular axis, children

## Abstract

The gut-vascular axis has emerged as a critical focus of research, with accumulating evidence suggesting distinct alterations in intestinal microbiota among patients with Kawasaki disease (KD). However, no systematic review to date has comprehensively characterized gut microbial dysbiosis in this population. This systematic review aimed to investigate changes in the composition of gut microbiota in KD patients. A comprehensive search of MEDLINE, EMBASE, Web of Science, and the Cochrane Library databases was conducted, and the study quality was assessed using the Newcastle-Ottawa Scale. All reported taxa were re-annotated according to the SILVA 138 database. Seven studies were enrolled in our review. In acute-phase KD, reduced *α*-diversity and significant *β*-diversity divergence were observed compared to healthy controls (HCs). Concurrently, taxa with potential short-chain fatty acid (SCFA)-linked protective functions showed diminished abundance, including *Bacteroidota, Bacteroides, Roseburia, Faecalibacterium, Blautia, Dialister, Lachnospira,* and *Prevotella*, while opportunistic pathogens such as *Enterococcus* were enriched in acute-phase KD cohorts. For non-acute KD patients, *β*-diversity remained distinct, with reduced abundance of the SCFA-producing genus *Blautia*. These findings suggest that gut microbiota dysbiosis may be associated with KD pathogenesis via immunomodulatory pathways, although the mechanistic insights remain to be elucidated.

**Systematic review registration:**

https://www.crd.york.ac.uk/PROSPERO/view/CRD420251148103

## 1 Background

Kawasaki disease (KD), first described by Japanese physician Tomisaku Kawasaki in 1967, is an acute febrile mucocutaneous lymph-node syndrome initially recognized as a self-limited vasculitis of unknown etiology (Kawasaki, 1967; Kawasaki et al., 1974; Jone et al., 2024). As an acute febrile illness, KD primarily affects children under 5 years of age, with marked variations in incidence reported across different countries (McCrindle et al., 2017). In Japan, the incidence reached 330.2 per 100,000 children in 2015, whereas in the United States, it ranged from 18 to 25 per 100,000 (Makino et al., 2015; Maddox et al., 2021). Coronary artery dilatation or aneurysm remains the most common complication, with approximately 25% of untreated KD patients developing coronary artery lesions (Seki and Minami, 2022). Furthermore, cardiovascular damage may persist into adulthood, placing individuals with a history of KD at increased risk for long-term cardiovascular events such as cardiomyopathy, myocardial ischemia, and premature atherosclerosis (Gordon et al., 2016; Gordon et al., 2009). Although emerging evidence suggests that the pathogenesis of KD is multifactorial, implicating genetic predisposition, antecedent infections, immune dysregulation, and environmental triggers, the precise mechanistic basis of this enigmatic vasculitis remains poorly understood (Lo, 2020; Rife and Gedalia, 2020).

The human gut microbiota, composed of over 100 trillion diverse microorganisms, including bacteria, viruses, fungi, and archaea, serves as a critical component of human physiology, contributing to nutrient digestion and absorption, maintaining intestinal mucosal barrier integrity, and regulating metabolic and immune systems (Durack and Lynch, 2019; Valdes et al., 2018; Natividad and Verdu, 2013; Adak and Khan, 2019). Accumulating evidence has demonstrated associations between gut microbiota dysregulation and a wide spectrum of diseases, including psychiatric, gastrointestinal, respiratory, and renal disorders, which has led to the conceptualization of gut-brain, gut-lung, and gut-kidney axes (Toader et al., 2024; Ekstedt et al., 2024; Karam et al., 2025; Tsuji et al., 2024; Ozcam and Lynch, 2024). Recently, the gut-vascular axis has emerged as a novel area of interest, with growing evidence suggesting that gut microbiota dysbiosis contributes to cardiovascular pathologies such as atherosclerosis, heart failure, hypertension, and abdominal aortic aneurysms (Flori et al., 2024; Li et al., 2024; Shen et al., 2025; Ge et al., 2024; Chui et al., 2024). KD is an acute febrile systemic vasculitis of early childhood, characterized by inflammation of small-to-medium-sized muscular arteries, particularly the coronary arteries. Consequently, KD is the leading cause of acquired heart disease among children in developed regions (Jone et al., 2024). In recent years, the characterization of gut microbiota in KD patients has emerged as a major research focus, with several studies reporting significant dysbiosis compared to healthy controls (HCs) (Chen et al., 2020; Khan et al., 2020; Shen et al., 2020; Fabi et al., 2022; Teramoto et al., 2023; Zeng et al., 2023; Han et al., 2024). Additionally, some studies suggest that the gut microbiota may play a significant role in KD development by modulating the host’s innate and adaptive immune responses and maintaining intestinal epithelial barrier function (Jena et al., 2025; Tao and Lang, 2024). These findings identify the gut microbiota as a promising therapeutic target for KD (Yang et al., 2025). However, existing studies on gut microbiota alterations in KD show considerable heterogeneity, and no comprehensive meta-analysis or systematic review has yet synthesized these findings. Therefore, this systematic review aims to summarize current evidence to delineate gut microbiota profiles in KD and provide a theoretical basis for the prevention, diagnosis, and treatment of KD.

## 2 Materials and methods

### 2.1 Search strategy

The present study was registered in the International Prospective Register of Systematic Reviews (PROSPERO) (CRD420251148103), and it was conducted in accordance with the Preferred Reporting Items for Systematic Reviews and Meta-Analyses (PRISMA) guidelines (Shamseer et al., 2015). The review followed the registered protocol, and no major deviations occurred during the process. A systematic literature search (last updated September 5, 2025) was performed in MEDLINE, EMBASE, Web of Science, and the Cochrane Library. The complete search strategies are provided in the Supplementary material, and gray literature was not included in the search strategy.

### 2.2 Eligibility criteria and study selection

Studies were selected based on the following inclusion criteria: (1) case–control studies comparing gut microbiota characteristics between KD patients and HCs; (2) use of high-throughput sequencing methods to analyze intestinal bacterial profiles. The exclusion criteria are as follows: (1) insufficient data for quantitative analysis (e.g., lack of specific microbiota abundance data); (2) culture-dependent methodologies, animal studies, reviews, study protocols, or letters; and (3) duplicate publications. Records retrieved from electronic databases were imported into EndNote (version X9.3.3) for automatic duplicate removal. Irrelevant records were removed through independent title and abstract screening by two reviewers. Full-text articles were then retrieved, and eligibility was assessed independently by two reviewers according to predefined inclusion and exclusion criteria. Discrepancies were resolved through discussion to reach a consensus. Any unresolved disagreements were adjudicated by a third reviewer. Inter-rater reliability for study inclusion was assessed using Cohen’s *κ*, yielding a value of 0.85, which indicated excellent agreement.

### 2.3 Data extraction and quality assessment

Data were independently and systematically extracted by two authors using a standardized data collection form. (1) General study characteristics: first author, publication year, country of origin, participant demographics (age and gender), sample size, and antibiotic exposure status and receipt of intravenous immunoglobulin (IVIG) treatment. (2) Gut microbiota features: *α*-diversity indices, *β*-diversity metrics, and relative taxonomic abundance. To ensure taxonomic consistency across studies employing different classification systems (e.g., SILVA, NCBI, and GTDB), all reported taxa were re-annotated according to the SILVA 138 database. When discrepancies were found, the SILVA-designated name was adopted, with the original name noted in parentheses for clarity. (3) Methodological details of microbiota collection and analysis, including sample type, storage conditions, DNA extraction, sequencing platform, targeted 16S rRNA regions, and reference database.

The quality of the included studies was assessed using the Newcastle–Ottawa Scale (NOS), which comprises eight items across three domains: selection, comparability, and exposure. Based on the NOS scoring system, studies were categorized as low quality (score 0–3), moderate quality (score 4–6), or high quality (score 7–9) (Lo et al., 2014).

## 3 Results

### 3.1 Study selection and quality assessment

**Figure 1:**
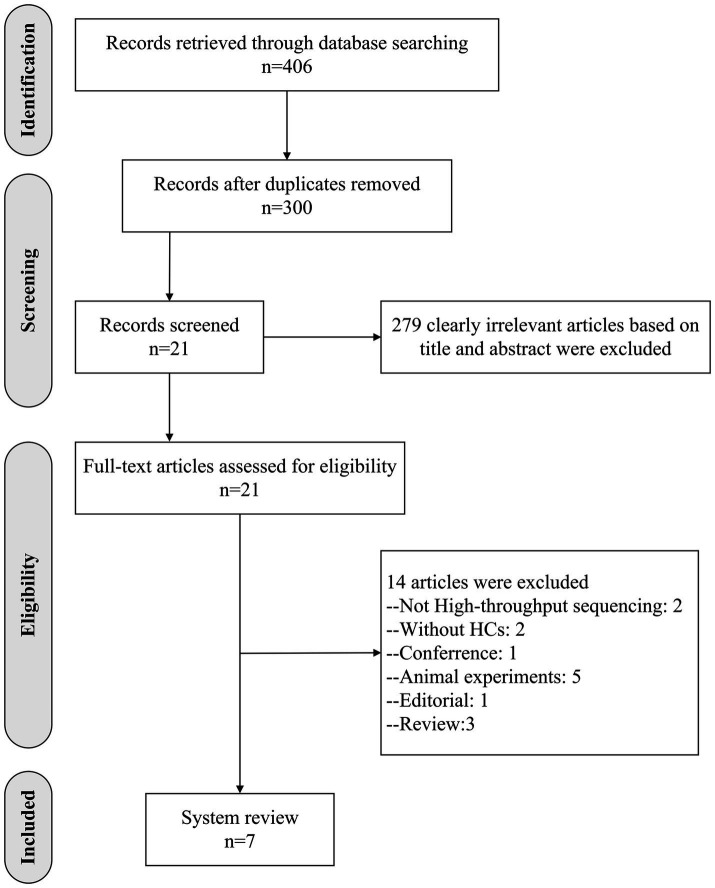
Study selection flow chart. HCs, healthy controls.

A total of 406 records were identified through database searches. After removing 106 duplicates and excluding 279 irrelevant articles based on titles and abstracts, 21 full-text articles were assessed for eligibility. Of these, 14 were excluded for the following reasons: non-high-throughput sequencing methods (*n* = 2), absence of HCs (*n* = 2), conference abstract (*n* = 1), animal studies (*n* = 5), editorial (*n* = 1), and review articles (*n* = 3). Finally, seven studies met the inclusion criteria and were included in the final analysis (Figure 1). Study quality was assessed using the NOS (Table 1); Six studies were rated as high quality (NOS scores 7–8), and one as moderate quality (score 6), indicating an overall satisfactory methodological standard.

**Table 1:** Quality assessment of the included studies using the Newcastle–Ottawa scale.

Study	Selection				Comparability	Exposure			Score
	Q1	Q2	Q3	Q4	Q5	Q6	Q7	Q8	
Chen et al. (2020)	1	1	1	1	1	1	1	1	8
Khan et al. (2020)	1	0	1	1	0	1	1	1	6
Shen et al. (2020)	1	1	1	1	1	1	1	1	8
Fabi et al. (2022)	1	1	1	0	1	1	1	1	7
Teramoto et al. (2023)	1	1	1	1	1	1	1	1	8
Zeng et al. (2023)	1	1	1	1	0	1	1	1	7
Han et al. (2024)	1	1	0	1	1	1	1	1	7

### 3.2 Study characteristics

This systematic review included seven studies published between 2020 and 2024 in English-language journals. All studies analyzed gut microbiota using stool samples. In six studies, samples were stored at −80 °C following collection, while one did not report storage conditions. 16S rRNA gene sequencing targeting the V4 or V3-V4 hypervariable regions was utilized in six studies, employing Illumina MiSeq or HiSeq platforms. Regarding sequence mapping, two used the GreenGenes database, two used the SILVA database (release v128 or v138), one study employed the Kyoto Encyclopedia of Genes and Genomes (KEGG) database (release 70.0), one used HUMANn3 with ChocoPhlAn and UniRef90 EC-filtered databases, and one did not specify the database used (Table 2).

**Table 2:** Technical information of the included studies.

Author (year)	Sample type	Sample storage	DNA extraction	Sequencing technique	Target 16S rRNA regions	Reference database
Chen et al. (2020)	Fecal samples	−80 °C	DNA Isolation Kit (MoBio, Carlsbad, CA, United States)	Illumina MiSeq platform (Illumina, San Diego, CA, United States)	V3-V4 hypervariable region of the 16S rRNA gene	Silva 16S rRNA database (Release v128)
Khan et al. (2020)	Fecal samples	NA	QIAamp DNA Stool Mini Kit (Qiagen, Hilden, Germany)	Illumina MiSeq platform (Illumina, Inc., CA, United States)	V4 hypervariable region of the 16S rRNA gene	GreenGenes database, RDPII, and NCBI
Shen et al. (2020)	Fecal samples	−80 °C	QIAamp DNA Stool Mini Kit (QIAGEN, Tokyo, Japan)	MiSeq-PE250	V3-V4 hypervariable region of the 16S rRNA gene	NA
Fabi et al. (2022)	Fecal samples	−80 °C	DNeasy Blood and Tissue Kit (QIAGEN, Hilden, Germany)	Illumina MiSeq platform (Illumina, San Diego, CA, United States)	V3-V4 hypervariable region of the 16S rRNA gene	GreenGenes database (May 2013 release)
Teramoto et al. (2023)	Fecal samples	−80 °C	NucleoSpin DNA Stool (MACHEREY-NAGEL, Dueren, Germany)	Illumina MiSeq platform (Illumina, San Diego, CA, United States)	V3-V4 hypervariable region of the 16S rRNA gene	Kyoto Encyclopedia of Genes and Genomes (KEGG) database (Release 70.0)
Zeng et al. (2023)	Fecal samples	−80 °C	E. Z. N. A. soil DNA Kit (Omega Biotek, Norcross, GA, United States)	Illumina MiSeq platform (Illumina, San Diego, CA, United States)	V3-V4 hypervariable region of the 16S rRNA gene	Silva 16S rRNA database (Release v138)
Han et al. (2024)	Fecal samples	−80 °C	Tiangen DNA Stool Mini Kit (Tiangen Biotech (Beijing) Co., Ltd., China)	Illumina NovaSeq 6,000 platform	NA	HUMANn3 with the ChocoPhlAn and UniRef90 EC filtered databases

NA, Not available.

**Figure 2:**
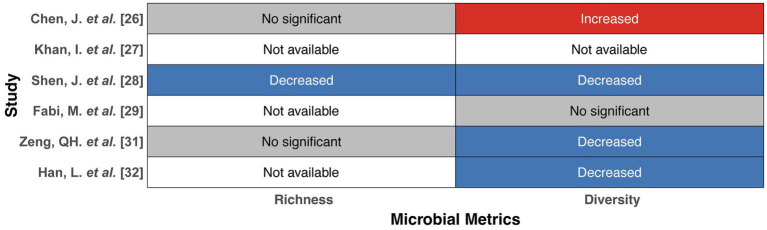
*α*-Diversity (richness and diversity) in patients with acute KD compared with HCs. This figure illustrates the changes in α-diversity (richness and diversity) of the gut microbiota in acute KD compared with HCs. Each row corresponds to a study, and the columns represent microbial diversity metrics (richness and diversity). Color coding indicates the direction of microbial change: red for increased values, blue for decreased values, gray for non-significant findings, and white for not available. KD, Kawasaki disease; HCs, healthy controls.

Geographically, five of the included studies were conducted in China (Chen et al., 2020; Khan et al., 2020; Shen et al., 2020; Zeng et al., 2023; Han et al., 2024), while the remaining two were conducted in Italy and Japan, respectively (Fabi et al., 2022; Teramoto et al., 2023). Among the seven studies, six compared gut microbiota profiles between acute KD patients and HCs (Chen et al., 2020; Khan et al., 2020; Shen et al., 2020; Fabi et al., 2022; Zeng et al., 2023; Han et al., 2024), whereas only two focused on non-acute KD patients, which were defined as those assessed ≥6 months after disease onset (Chen et al., 2020; Teramoto et al., 2023). Participant characteristics are summarized in Tables 3, 4. As shown in Table 5, *α*- and *β*-diversity, as well as bacterial composition at various taxonomic levels, were compared between acute KD patients and HCs. α-diversity reflects microbial richness and diversity, typically assessed using various indices. Specifically, richness was evaluated using the Chao1, ACE, and Sobs indices. Bacterial diversity was assessed using the Shannon and Simpson indices. Among the included studies, 1 (1/6) reported reduced richness indices in acute KD patients compared with HCs, 3 (3/6) reported decreased diversity indices, whereas 1 (1/6) reported increased diversity (Figure 2). β-diversity, representing overall differences in community structure, was assessed using various statistical approaches, including principal coordinate analysis (PCoA), principal component analysis (PCA), Bray–Curtis dissimilarity, non-metric multidimensional scaling (NMDS), and partial least squares discriminant analysis (PLS-DA). Overall, 5 (5/6) studies reported significant differences in β-diversity between acute KD patients and HCs.

**Table 3:** Characteristics of acute KD patients and HCs in the included studies.

Author (year)	Country	Patients		Acute KD vs. HCs		
		IVIG	Antibiotic treatment	Participants numbers (acute KD vs. HCs)	Age* (range in years)	Male ratio
Chen et al. (2020)	China	NA	NO	30 vs. 30	2.36 ± 1.39 vs. 2.45 ± 1.27 ^**^	57% vs. 53% ^***^
Khan et al. (2020)	China	NO	YES (3/5)	NA^+^	2.17 (1–3) vs. NA	NA
Shen et al. (2020)	China	NO	NO	48 vs. 46	2.5 ± 1.6 vs. 3.0 ± 1.7 ^**^	50% vs. 54%^***^
Fabi et al. (2022)	Italy	NO	NA	13 vs. 12	2.58 (1.21–3.58) vs. 2.5 (1.25–5.65)	77% vs. 58%
Zeng et al. (2023)	China	NA	NO	5 vs. 4	2.33 (1.75–3) vs. 2.88 (1.92–3.25) ^**^	40% vs. 50%^***^
Han et al. (2024)	China	NO	NA	NA^#^	NA	NA

*The age of the participants was expressed as mean ± standard deviation or median (min to max of age). **The two groups were matched for age. ***The two groups were matched for sex. No symbols in the age or sex columns indicate that the original study did not report matching for those characteristics. ^**+**^Thirteen samples were collected from five patients during the acute phase of KD. Three samples were obtained from normal volunteers; however, the exact number of volunteers was not reported. ^**#**^Sixty-two samples were collected from 95 KD patients before IVIG treatment. The exact number of volunteer controls contributing to the 62 control samples was not reported. NA, Not available; KD, Kawasaki disease; HCs, Healthy controls; IVIG, Intravenous immunoglobulin.

**Table 4:** Characteristics of non-acute KD patients and HCs in the included studies.

Author (year)	Country	Antibiotic treatment	Non-acute KD vs. HCs		
			Participants numbers (Non-acute KD vs. HCs)	Age* (range in years)	Male ratio
Chen et al. (2020)	China	No	30 vs. 30	6 months after the onset age Onset age: 2.36 ± 1.39 vs. 2.45 ± 1.27	57% vs. 53%^**^
Teramoto et al. (2023)	Japan	No	26 vs. 57	1 year after the onset age Onset age: 2.71 (2.08–3.77) vs. 3.00 (2.04–4.00)	46% vs. 61%^**^

*The age of the participants was expressed as mean ± standard deviation or median (min to max of age). **The two groups were matched for sex. No symbols in the age column indicate that the original study did not report matching for that characteristic. KD, Kawasaki disease; HCs, Healthy controls.

**Table 5:** Alterations in gut microbiota in patients with acute KD compared with HCs.

Author	α-diversity	β-diversity	Gut microbiota characteristic
Chen et al. (2020)	ACE index: No significant difference. Simpson index: Increased.	Significant difference (PCoA).	Genus level: Increased: Staphylococcus; Butyricimonas; Lactococcus; Helicobacter; Acinetobacter; Enterococcus; Decreased: Agathobacter; Enterobacter; Prevotella_9; Blautia; Dialister; Clostridium_sensu_stricto_1; Eubacterium_eigens_group; Lachnoclostridium; Roseburia; Megasphaera; Lachnospira; Fusicatenibacter; Romboutsia; Citrobacter; Eubacterium_oxidoreducens_group; Eubacterium_ruminantium_group; Holdemanella; Paeniclostridium; Tunicibacter; Ruminococcaceae_UCG_005; Terrisporobacter
Khan et al. (2020)	NA	Significant difference (PCA).	Phylum level: Increased: Pseudomonadota (Proteobacteria); Verrucomicrobiota (Verrucomicrobia); Decreased: Bacteroidota (Bacteroidetes); Mycoplasmatota (Tenericutes)
Shen et al. (2020)	Chao1 index and Shannon index: Decreased.	Significant difference (PLS-DA).	Phylum level: Decreased: Bacteroidota (Bacteroidetes); Genus level: Increased: Enterococcus; Decreased: Bacteroides; Dorea
Fabi et al. (2022)	Shannon index: No significant difference.	Significant difference (PCoA).	Genus level: Decreased: Anaerostipes; Lachnospira; Blautia; Roseburia; Ruminococcus; Faecalibacterium; Dialister
Zeng et al. (2023)	Sobs index: No significant difference. Shannon index: Decreased.	Significant difference (PLS-DA and NMDS).	Phylum level: Increased: Bacillota (Firmicutes); Actinobacteriota (Actinobacteria); Pseudomonadota (Proteobacteria); Decreased: Bacteroidota; Genus level: Increased: Enterococcus; Decreased: Bacteroides; Faecalibacterium; Clostridium_sensu_stricto_1; Parabacteroides; Anaerostipes; Ruminococcus_torques_group; Alistipes; Lachnospira; Eubacterium_eligens_group; Hungatella; Roseburia; Campylobacter; Subdoligranulum; norank_f__Eubacterium_coprostanoligenes_group; Agathobacter; Lachnospiraceae_UCG-004; Flavonifractor; Prevotella; unclassified_f__Lachnospiraceae; Romboutsia; Intestinibacter; Eubacterium_nodatum_group; norank_f__Lachnospiraceae; Oscillibacter; norank_f__Ruminococcaceae; Holdermania; Ruminococcus_gauvreauii_group; Bilophila; Colidextribacter; Oscillospira; Coprobacillus; unclassified_f__Oscillospiraceae; unclassified_f__Ruminococcaceae
Han et al. (2024)	Shannon and Simpson indices: Decreased.	No Significant difference.	Genus level: Increased: Bifidobacterium; Enterococcus; Finegoldia; *Finegoldia magna*; *Enterococcus avium*; Decreased: Bacteroides

All reported taxa were re-annotated according to the SILVA 138 database. In cases of discrepancy, the SILVA-designated name was adopted, and the original name was retained in parentheses for clarity. KD, Kawasaki disease; HCs, Healthy controls; NA, Not available; PCoA, Principal coordinates analysis; PCA, Principal component analysis; PLS-DA, Partial least squares discriminant analysis; NMDS, Nonmetric multidimensional scaling.

Furthermore, α- and β-diversity metrics, along with bacterial composition at various taxonomic levels, were compared between non-acute KD patients and HCs, as summarized in Table 6. One study reported an increased richness index and reduced diversity of gut microbiota in non-acute KD patients, whereas another study demonstrated no significant difference in the diversity of gut microbiota between non-acute KD patients and HCs. For β-diversity, both studies reported significant differences between non-acute KD patients and HCs.

**Table 6:** Alterations in gut microbiota in patients with non-acute KD compared with HCs.

Author	α-diversity	β-diversity	Gut microbiota characteristic
Chen et al. (2020)	Ace index: Increased. Simpson index: Decreased.	Significant difference (PCoA).	NA
Teramoto et al. (2023)	Shannon and Simpson indices: No significant difference.	Significant difference (PCoA).	Genus level: Decreased: Blautia;

KD: Kawasaki disease; HCs: Healthy controls; NA: Not available; PCoA: Principal coordinates analysis. All taxa were re-annotated according to the SILVA 138 database. In cases of discrepancy, the SILVA-designated name was adopted, and the original name was retained in parentheses for clarity.

**Figure 3:**
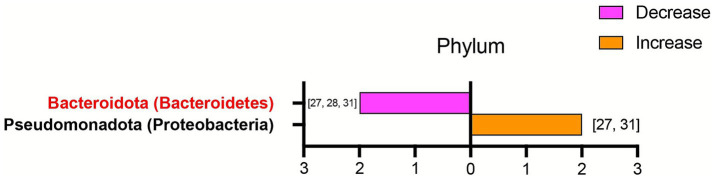
Alterations in gut microbiota at the phylum level in patients with acute KD compared with HCs. This figure presents phylum-level bacteria reported in more than one included study, with a total of three studies contributing data. The horizontal axis shows the number of studies reported for each corresponding phylum. The first taxon, highlighted in red, represents a short-chain fatty acid-producing bacterium. The orange bar represents the corresponding bacterium that increased in the acute KD group. The purple bar represents the corresponding bacterium that decreased in the acute KD group. The specific bacteria are listed beside the bars. All taxa were re-annotated according to the SILVA 138 database. In cases of discrepancy, the SILVA-designated name was adopted, and the original name was retained in parentheses for clarity. KD, Kawasaki disease; HCs, healthy controls.

**Figure 4:**
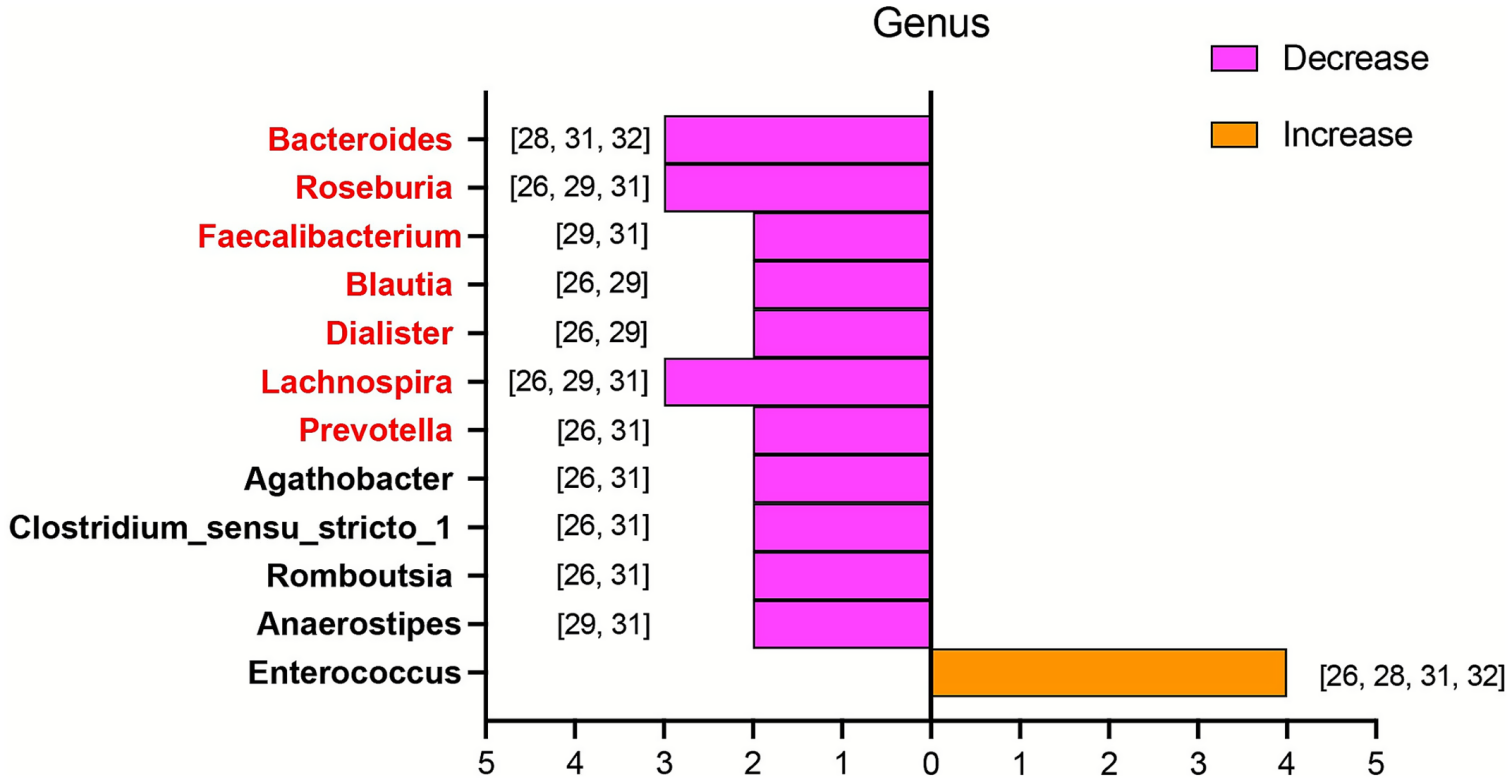
Alterations in gut microbiota at the genus level in patients with acute KD compared with HCs. This figure presents genus-level bacteria reported in more than one included study, with a total of five studies contributing data. The horizontal axis shows the number of studies reporting each genus. The first seven taxa, highlighted in red, represent short-chain fatty acid-producing bacteria. The orange bar represents the corresponding bacterium that increased in the acute KD group. The purple bar represents the corresponding bacteria that decreased in the acute KD group. The specific bacteria are listed beside the bars. All taxa were re-annotated according to the SILVA 138 database. In cases of discrepancy, the SILVA-designated name was adopted, and the original name was retained in parentheses for clarity. KD, Kawasaki disease; HCs, healthy controls.

Representative taxa in acute KD patients were summarized at the phylum and genus levels across included studies (Table 5). At the phylum level, the relative abundance of *Pseudomonadota (Proteobacteria)* was elevated in two studies, whereas *Bacteroidota (Bacteroidetes)* showed a decreased trend (Figure 3). At the genus level, 12 genera were recurrently identified across multiple studies (Figure 4). Among these, *Bacteroides, Roseburia, Faecalibacterium, Blautia, Dialister, Lachnospira, Prevotella, Agathobacter, Clostridium_sensu_stricto_1, Romboutsia,* and *Anaerostipes* were reduced in patients with acute KD. Conversely, only *Enterococcus* was consistently enriched in acute KD patients. Additionally, only two studies focused on non-acute KD patients, and one of them reported that, compared with HCs, the abundance of Blautia was decreased (Table 6).

## 4 Discussion

Accumulating evidence indicates that the gut microbiota plays a crucial role in human health and is associated with the development of many diseases by modulating physiological processes (Kim et al., 2024). The gut-vascular axis has recently attracted increasing research attention, with several studies establishing correlations between gut microbiota composition and cardiovascular disorders (Flori et al., 2024). KD, an acute systemic vasculitis primarily affecting small- to medium-sized arteries, predominantly occurs in children. A subset of KD patients presents with gastrointestinal manifestations, which are now recognized as potential contributors to the disease’s clinical progression (Tao and Lang, 2024; Fabi et al., 2018). Notably, Esposito *et al*. proposed a potential causal relationship between gut microbiota dysbiosis and the pathogenesis of KD (Esposito et al., 2019). In response, a growing number of studies have attempted to characterize alterations in gut microbiota in KD; however, the reported findings remain inconsistent across studies. To our knowledge, this systematic review is the first to comprehensively characterize the gut microbiota composition in KD, based on rigorous quality assessment, standardized data extraction, and analysis.

The present systematic review demonstrated that the composition of the gut microbiota in KD patients varies with disease progression. Regarding gut microbial diversity, KD patients exhibited significant differences compared with HCs. Specifically, *α*-diversity, reflecting microbial richness and diversity, was decreased in KD patients in three studies (Shen et al., 2020; Zeng et al., 2023; Han et al., 2024), while recovery of α-diversity was observed during the non-acute phase (Teramoto et al., 2023). Decreased α-diversity has likewise been reported in other cardiovascular and vascular inflammatory conditions, including Immunoglobulin A vasculitis, antineutrophil cytoplasmic antibody-associated vasculitis, hypertension, and coronary artery disease (Wang et al., 2018; Yu et al., 2022; Cai et al., 2023; Choroszy et al., 2022). Regarding *β*-diversity, six of the seven included studies reported distinct overall microbial community structures in KD patients, including those in the non-acute phase, compared with HCs, consistent with findings from other studies characterizing gut microbiota alterations in cardiovascular disease (Teramoto et al., 2023; Cheng et al., 2022; Qin et al., 2022; Zhang et al., 2021).

As for alterations in specific microbiota in acute KD patients, although no completely consistent results were identified across the included studies, two major groups of bacteria with potential relevance to the development of KD were observed. The first group consisted of *Bacteroidota, Bacteroides, Roseburia, Faecalibacterium, Blautia, Dialister, Lachnospira, and Prevotella,* which are recognized as short-chain fatty acid (SCFA)-producing bacteria (Yang et al., 2020; Akhtar et al., 2022; Liu et al., 2021; Stoeva et al., 2021). SCFAs are microbial metabolites derived from the fermentation of non-digestible dietary fibers and provide multiple beneficial effects on the host, which include maintaining gut ecological homeostasis, modulating metabolic pathways, promoting antimicrobial activity, and regulating both intestinal and systemic immune responses (Akhtar et al., 2022; Facchin et al., 2024). Accumulating clinical and animal evidence indicates that SCFAs are associated with the development of many cardiovascular diseases, and supplementation with SCFAs has been shown to regulate blood pressure, alleviate atherosclerosis, and inhibit the progression of abdominal aortic aneurysms (Kaye et al., 2020; Bartolomaeus et al., 2019; Tian et al., 2022; Verhaar et al., 2020). Emerging evidence suggests that SCFAs may influence the pathogenesis of KD through three primary mechanisms. First, SCFAs enhance intestinal barrier integrity by stimulating the secretion of mucins and antimicrobial peptides, which are essential for maintaining the gut’s microbial and chemical barriers. Second, SCFAs help preserve microbial homeostasis by suppressing pathogen colonization and promoting the growth of beneficial commensal bacteria (Zhang et al., 2022). Therefore, reduced SCFA levels may impair both the physical and biological barriers of the intestine in KD patients, thereby permitting translocation of pathogens and microbial-derived toxins into the bloodstream. This process may subsequently stimulate local and systemic inflammatory responses by upregulating cytokines such as tumor necrosis factor-*α* (TNF-α), interferon-*γ* (IFN-γ), and interleukin-1β (IL-1β), ultimately exacerbating disease progression in KD (Hemmati et al., 2023; Meyer et al., 2023). Third, SCFAs may influence the progression of KD by modulating host inflammatory activation through multiple immunological pathways. An animal study using a KD mouse model demonstrated that butyrate, a key SCFA, significantly reduced the expression of pro-inflammatory cytokines such as IL-1β, IL-8, and TNF-α and inhibited the activation of the JNK, ERK1/2, and p38 MAPK signaling pathways. These anti-inflammatory effects of butyrate ultimately attenuated coronary artery lesions by reducing inflammatory cell infiltration into vascular tissues (Wang et al., 2023). Moreover, SCFAs may influence the development of KD by regulating the differentiation and maturation of T cell subsets. A growing body of evidence suggests that SCFAs facilitate the differentiation of naïve T cell precursors into regulatory T cells (Tregs), while simultaneously suppressing the development of pro-inflammatory T helper 17 (Th17) cells (Asarat et al., 2016; Guo et al., 2015). It has been postulated that reduced SCFA levels, resulting from gut microbiota dysbiosis, may disrupt the Th17/Treg balance, thereby being linked to the heightened inflammatory responses observed in KD patients (Luo et al., 2017). In the present review, more than two included studies reported a significant decrease in the richness of SCFA-producing bacteria in acute KD patients compared with HCs. Based on these findings, we speculate that diminished taxa with potential SCFA-linked protective functions may be associated with the exacerbation of KD by enhancing both local and systemic inflammation through activation of pro-inflammatory signaling pathways, increased cytokine secretion, and perturbation of Th17/Treg homeostasis.

Among the above-mentioned SCFA-producing bacteria, *Prevotella* warrants particular attention due to its paradoxical role in inflammation. As an SCFA-producing bacterium, *Prevotella* exerts anti-inflammatory effects through multiple mechanisms, as previously described. Conversely, it has also been identified as a pro-inflammatory bacterium, with elevated abundance observed at mucosal surfaces in various inflammatory conditions, including rheumatoid arthritis, ankylosing spondylitis, HIV infection, and respiratory viral infections (Tsetseri et al., 2023; Wen et al., 2017; Mingjun et al., 2022; Tsang et al., 2020; Tamanai-Shacoori et al., 2022). Numerous *in vitro* studies have demonstrated that *Prevotella*, compared with commensal oral bacteria, exhibits a stronger ability to induce pro-inflammatory cytokines such as IL-6, IL-8, and TNF-α (Cury et al., 2013; Horst et al., 2009; Ji et al., 2007). Further, Maeda et al. reported that gut microbiota dominated by *Prevotella* may upregulate intestinal Th17 cell populations, thereby enhancing the host’s inflammatory response (Maeda et al., 2016). In the present systematic review, we found that the richness of *Prevotella* was decreased in acute KD patients compared with HCs in two included studies. As an SCFA-producing bacterium, a reduction in *Prevotella* may diminish SCFA availability and thereby be associated with the progression of KD. Conversely, given its recognized pro-inflammatory properties, a decrease in *Prevotella* could theoretically attenuate inflammatory responses and thus impede disease progression. Thus, we speculate that although *Prevotella* has been implicated in pro-inflammatory processes, its anti-inflammatory functions may be more prominent during the acute phase of KD. Consequently, a reduction in *Prevotella* abundance might exacerbate systemic inflammation and facilitate the progression of KD.

As a Gram-positive, facultative anaerobic bacterium, *Enterococcus* colonizes the host’s gastrointestinal tract and is recognized as an opportunistic pathogen that can cause urinary tract infections, hepatobiliary sepsis, endocarditis, and bacteremia (Fisher and Phillips, 2009). Accumulating evidence indicates that *Enterococcus* predominates in patients with various cardiovascular diseases, including atrial fibrillation, heart failure, and Henoch–Schönlein purpura (Fabi et al., 2022; Zuo et al., 2019; Zhang et al., 2023). Stein–Thoeringer *et al*. reported that *Enterococcus*-derived metabolites, including arachidonic acid derivatives, 20-hydroxy-leukotriene B4, and leukotriene F4, may impair the intestinal barrier and subsequently stimulate host inflammatory responses (Stein-Thoeringer et al., 2019). Steck N. et al. also proposed that *Enterococcus faecalis* compromises the gut epithelial barrier by secreting gelatinase, thereby being linked to intestinal inflammation (Steck et al., 2011). Furthermore, *Enterococcus* exhibits a strong capacity for biofilm formation, which may stimulate the host to produce various superantigens, thereby inducing inflammatory responses (Guiton et al., 2010). Our results showed a significant increase in the abundance of *Enterococcus* in acute KD patients compared with HCs. This predominant *Enterococcus* may initiate or exacerbate host inflammation by disrupting the intestinal barrier integrity and producing superantigens, thereby being associated with the development of KD. The precise mechanisms underlying these associations warrant further investigation.

There is several limitations in this review. First, due to methodological heterogeneity and limited gut microbiota data across the included studies, a meta-analysis could not be performed; thus, we conducted a systematic review instead. Moreover, several studies lacked detailed reporting of participant characteristics, such as group size and age or sex matching. These omissions may introduce bias and limit comparability between the KD and control groups. Second, studies published in Chinese databases were not searched, which may have introduced language bias. Third, only seven studies were included, the majority of which were conducted in China, where KD has a relatively high incidence and distinctive dietary patterns. Notably, the composition of the gut microbiota is shaped by geographic region and dietary habits (Yu et al., 2025; Abdill et al., 2025), which may limit the generalizability of findings from studies conducted predominantly in East Asia. Fourth, variability in microbiota analysis methodologies, including DNA extraction protocols, sequencing platforms, and bioinformatics pipelines, was observed across studies, potentially affecting microbial composition results. Fifth, some observational studies did not fully account for the effects of antibiotics and IVIG on gut microbiota. Only three studies explicitly stated that participants had not taken antibiotics, and only four confirmed that IVIG had not been administered before sampling. However, it is well established that antibiotics can disrupt gut microbial communities, specifically by reducing diversity and altering community structure (Fishbein et al., 2023). Conversely, evidence regarding the impact of IVIG on gut microbiota is inconsistent: one clinical study found that IVIG administration did not significantly alter gut microbiome composition in the short term (Svačina et al., 2023), whereas animal models have reported IVIG-related microbial changes (Ramakrishna et al., 2020). Therefore, the potential confounding effects of antibiotic and IVIG administration should not be overlooked. To summarize, more rigorously designed investigations that account for confounding factors such as sample size, geographic location, and antibiotics or IVIG use are required to illuminate the relationship between the intestinal microbiota and the development of KD.

## 5 Conclusion

This review revealed significant alterations in gut microbiota among KD patients, characterized by a reduction in taxa with potential SCFA-linked protective functions and an enrichment of opportunistic pathogens. It is hypothesized that these alterations in the gut microbiota may be associated with KD pathogenesis by modulating host immune responses through the gut–vascular axis, highlighting the potential of microbiota-targeted strategies for KD prevention and treatment. However, interpretation should be cautious due to small sample sizes, regional concentration of studies in East Asia, and methodological heterogeneity in sequencing platforms and taxonomic annotation.

## Data Availability

The original contributions presented in the study are included in the article/Supplementary material, further inquiries can be directed to the corresponding authors.
