# Supplementary material for: The characteristics of the gut microbiota in patients with Kawasaki disease: a systematic review
Source: Front Microbiol. 2025 Dec 18;16:1715478. doi: 10.3389/fmicb.2025.1715478 (PMC12756895; doi:10.3389/fmicb.2025.1715478)
Supplement: Supplementary file 1 [file Data_Sheet_1.docx]

The specific search strategies of the present study

The specific search strategies in Medline database are as follows:

1. Lymphatic Diseases.af.

2. Mucocutaneous Lymph Node Syndrome.af.

3. ((kawasaki adj1 disease) or (kawasaki adj1 syndrome)).af.

4. 1 or 2 or 3

5. Gastrointestinal Microbiome.af.

6. gut microbiota.ab,kw,ti.

7. gut microbiome.ab,kw,ti.

8. gastrointestinal microbiome.ab,kw,ti.

9. gastrointestinal microbiota.ab,kw,ti.

10. microbiota.ab,kw,ti.

11. microbiome.ab,kw,ti.

12. microflora.ab,kw,ti.

13. flora.ab,kw,ti.

14. 5 or 6 or 7 or 8 or 9 or 10 or 11 or 12 or 13

15. 4 and 14

The specific search strategies in Embase database are as follows:

1. Lymphatic Diseases.af.

2. Mucocutaneous Lymph Node Syndrome.af.

3. ((kawasaki adj1 disease) or (kawasaki adj1 syndrome)).af.

4. 1 or 2 or 3

5. Gastrointestinal Microbiome.af.

6. gut microbiota.ab,kw,ti.

7. gut microbiome.ab,kw,ti.

8. gastrointestinal microbiome.ab,kw,ti.

9. gastrointestinal microbiota.ab,kw,ti.

10. microbiota.ab,kw,ti.

11. microbiome.ab,kw,ti.

12. microflora.ab,kw,ti.

13. flora.ab,kw,ti.

14. 5 or 6 or 7 or 8 or 9 or 10 or 11 or 12 or 13

15. 4 and 14

The specific search strategies in Cochrane database are as follows:

1. Lymphatic Diseases

2. Mucocutaneous Lymph Node Syndrome

3. ((kawasaki adj1 disease) or (kawasaki adj1 syndrome))

4. 1 or 2 or 3

5. Gastrointestinal Microbiome

6. gut microbiota

7. gut microbiome

8. gastrointestinal microbiome

9. gastrointestinal microbiota

10. microbiota

11. microbiome

12. microflora

13. flora

14. 5 or 6 or 7 or 8 or 9 or 10 or 11 or 12 or 13

15. 4 and 14

The specific search strategy in Web of science database is as follows:

1. Mucocutaneous Lymph Node Syndrome (TS) or Lymphatic Diseases (TS) or kawasaki disease (TS) or Kawasaki syndrome (TS)

2. gut microbiota (TS) or gut microbiome (TS) or gastrointestinal microbiome (TS) or gastrointestinal microbiota (TS) or microbiota (TS) or microbiome (TS) or microflora (TS) or flora (TS)

3.#2 and #1
